# Seroprevalence report on tick-borne encephalitis virus and Crimean-Congo hemorrhagic fever virus among Malaysian’s farm workers

**DOI:** 10.1186/s12889-015-1901-4

**Published:** 2015-07-24

**Authors:** Munirah Mohd Shukri, Kai Ling Kho, Masoumeh Ghane Kisomi, Rafidah Lani, Suria Marlina, Siti Fatimah Muhd Radzi, Sun Tee Tay, Li Ping Wong, Awang Bulgiba Awang Mahmud, Quaza Nizamuddin Hassan Nizam, Sazaly Abu Bakar, Keivan Zandi

**Affiliations:** Department of Medical Microbiology, Faculty of Medicine, University of Malaya, Kuala Lumpur, Malaysia; Department of Social and Preventive Medicine, Faculty of Medicine, University of Malaya, Kuala Lumpur, Malaysia; Department of Veterinary Services, Federal Government Administration Centre, Putrajaya, Malaysia

**Keywords:** Tick borne viruses, TBEV, CCHFV, Seroprevalence, Farmer workers, Malaysia

## Abstract

**Background:**

Tick-borne encephalitis virus (TBEV) and Crimean-Congo haemorrhagic fever virus (CCHFV) are important tick-borne viruses. Despite their wide geographical distribution and ease of acquisition, the prevalence of both viruses in Malaysia is still unknown. This study was conducted to determine the seroprevalence for TBEV and CCHFV among Malaysian farm workers as a high-risk group within the population.

**Methods:**

We gave questionnaires to 209 farm workers and invited them to participate in the study. Eighty-five agreed to do so. We then collected and tested sera for the presence of anti-TBEV IgG (immunoglobulin G) and anti-CCHFV IgG using a commercial enzyme-linked immunosorbent assay (ELISA) kit. We also tested seroreactive samples against three other related flaviviruses: dengue virus (DENV), West Nile virus (WNV) and Japanese encephalitis virus (JEV) using the ELISA method.

**Results:**

The preliminary results showed the presence of anti-TBEV IgG in 31 (36.5 %) of 85 sera. However, when testing all the anti-TBEV IgG positive sera against the other three antigenically related flaviviruses to exclude possible cross reactivity, only five (4.2 %) sera did not show any cross reactivity. Interestingly, most (70.97 %) seropositives subjects mentioned tick-bite experience. However, there was no seroreactive sample for CCHFV.

**Conclusions:**

These viruses migrate to neighbouring countries so they should be considered threats for the future, despite the low seroprevalence for TBEV and no serological evidence for CCHFV in this study. Therefore, further investigation involving a large number of human, animal and tick samples that might reveal the viruses’ true prevalence is highly recommended.

## Background

Ticks are important and prevalent vectors for several animal and human infectious diseases, carrying harmful pathogens such as Borrelia spp, Rickettsia spp, Babesia spp, and various viruses including TBEV and CCHFV. TBEV is a member of the genus Flavivirus within the Flaviviridae family. This etiologic agent of tick-borne encephalitis can cause a potentially fatal neurological infection affecting the human central nervous system. TBEV has three subtypes: European (TBEV-EU), Far Eastern (TBEV-Fe) and Siberian (TBEV-Sib) [1]. *Ixodes ricinus* is the main vector for TBEV-EU, while the other two subtypes are transmitted mainly by I.persulcatus [2]. The vector facilitates virus transmission to other vertebrates, which also act as a reservoir for the virus. Ixodes ticks acquire TBEV by feeding on viraemic animals, especially small rodents that serve as main vertebrate hosts and virus reservoirs [3]. The virus will be carried by infected ticks for life and maintained transstadially from one life stage to another. TBEV also transmits transovarially from parents to their progeny. Typically, humans are the accidental dead-end host for TBEV and a tick bite is the main route for the virus to enter the human body. A secondary mode of transmission is consuming unpasteurized milk and milk products from viraemic livestock [4]. Several reports show that TBEV is not only endemic in European countries, but also in Asian countries such as China, Japan, Mongolia, Kazakhstan and Kyrgyzstan [1, 5].

CCHFV is a tick-borne virus belonging to the genus Nairovirus in the Bunyaviridae family and a causative agent for a deadly viral haemorrhagic fever. Despite a history of isolation from many genera and species of ticks, the main vector for this virus is a tick from the Hyalomma genus [6]. Similar to TBEV, transovarial and transstadial transmission of CCHFV have been reported, and small rodents are its major reservoirs. The CCHFV life cycle involved tick and a variety of wild and domestic vertebrates, including large mammals like cattle, sheep and goat [7], where infected mammals develop viraemia without visible sign [8]. In addition to transmission through tick bites, crushing an infected ticks and direct contact with tissue or body fluids of viraemic-infected individuals and animals is an alternative route for viral transmission to humans [9]. CCHFV is endemic in over 30 Eurasian and African countries. Therefore, it is a widely distributed virus with the broadest geographical distribution among all tick-borne viruses [7, 10]. Human death rate due to CCHFV infection ranges from 5 to 80 % [11].

Although TBEV and CCHFV are not endemic in Malaysia, circulation of other vector borne viruses are reported in this country, including DENV, WNV, JEV and the Langat virus (LGTV) [12–15]. Flaviviruses commonly share one or more antigenic sites among their members. However, they can be differentiated from each other using several tests, the most commonly being used is the virus neutralization test.

Seroepidemiological studies related to the prevalence of TBEV and CCHFV have been widely performed, especially among high-risk groups including farmers. To date, there is no reported case or outbreak of those viruses in Malaysia, but there was a study by Thayan and colleagues on screening for TBEV antibodies [16] among Malaysian patients with encephalitis. They did not find any seroreactivity among any tested samples. However, our unpublished data revealed a prevalence of 17.6 % anti-TBEV IgG when we screened within the Orang Asli population who were living deep in the forests of Peninsular Malaysia. Since zoonotic infections are potential occupational hazards among high-risk groups, including farmers and animal farm workers, and to show the current situation in Malaysia, this study has been designed and conducted to investigate the seroprevalence for anti-TBEV and anti-CCHFV IgG among Malaysian farm workers as one of the most important high-risk populations.

## Methods

### Ethic statements

The study’s protocol was approved by the Ethics Committee Universiti Malaya Medical Centre (MEC Ref. 824.11 and MEC Ref. 944.20). Participation in the study was entirely voluntary. Potential participants were briefed on the project and given sufficient time for consideration. All subjects gave written informed consent for inclusion before they participated in the study. The blood samples were handled with strict anonymity. All participants gave written consent for the samples to be used after anonymisation.

### Population and sample collection

**Fig. 1:**
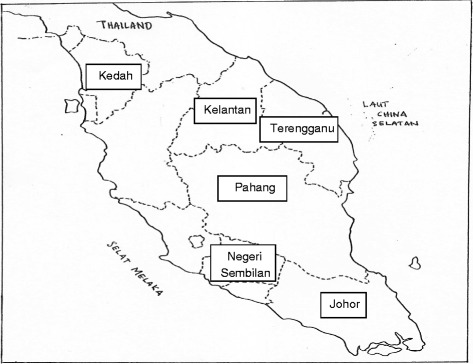
Farms located in different regions of Peninsular Malaysia. The figure shown the location of farms where serum samples were collected. 2 farms located in Negeri Sembilan, another 2 farms in Pahang, while one farm each in Kedah, Kelantan, Terengganu and Johor

Eleven cattle and goat farms in Peninsular Malaysia were identified from information from the Department of Veterinary Services (DVS), Ministry of Agriculture and Agro-based Industry, Malaysia. All were contacted and invited to take part in the project. Eight of the 11 farms agreed to participate in the study. These eight farms were located in different regions of Peninsular Malaysia (Fig. 1). Seven farms practised rotational grazing; only one had zero grazing, where fodder was carried to the cattle unit. Two of the eight farms applied acaricides once every 6 months, one farm applied them every 2 months, and another two farms applied them monthly. Three farms applied acaricides only when tick infestation became a problem. We gave questionnaires to all 209 employees. Despite incentives, only 85 workers agreed to participate in our study. The participants were 83.5 male and 16.5 % female, and aged between 20 and 60 years; 39 (45.8 %) were between 20 and 35, 23 (27.1 %) between 36 and 50, and 23 (27.1 %) between 51 and 60. We collected serum samples from September 2012 to February 2013, helped by an experienced medical assistant. We documented age, sex, tick-bite experience and vaccination history for JEV for each participant.

### Serological studies

We analysed serum samples for the presence of anti TBEV IgG and anti-CCHFV IgG using the commercial TBEV IgG ELISA kit (Abnova® Corporation, Taiwan) and VectoCrimean-CHF-IgG kit (Vector-Best, Novosibirsk, Russia) in accordance with the manufacturers’ instructions. We then tested positive sera for the presence of anti-DENV, anti-WNV and anti-JEV IgG antibodies to exclude cross reactivity with the positive sera for TBEV IgG. Finally, we performed cross reactivity tests using the DENV IgG ELISA kit (Abnova® Corporation, Taiwan), the WNV IgG capture DxSelect ELISA kit (Focus Diagnostics, Cypress, USA) and the JEV IgG ELISA kit (Inbios International, USA).

### Statistical analysis

All data were analysed using the Fisher exact test for analysis of contingency table. We determined the correlations between seropositivity and different age groups using Spearman non-parametric correlation. All statistical analyses were conducted using GraphPad Prism 6 (GraphPad Software, Inc.), where *p* < 0.05 is considered significant.

## Results

**Fig. 2:**
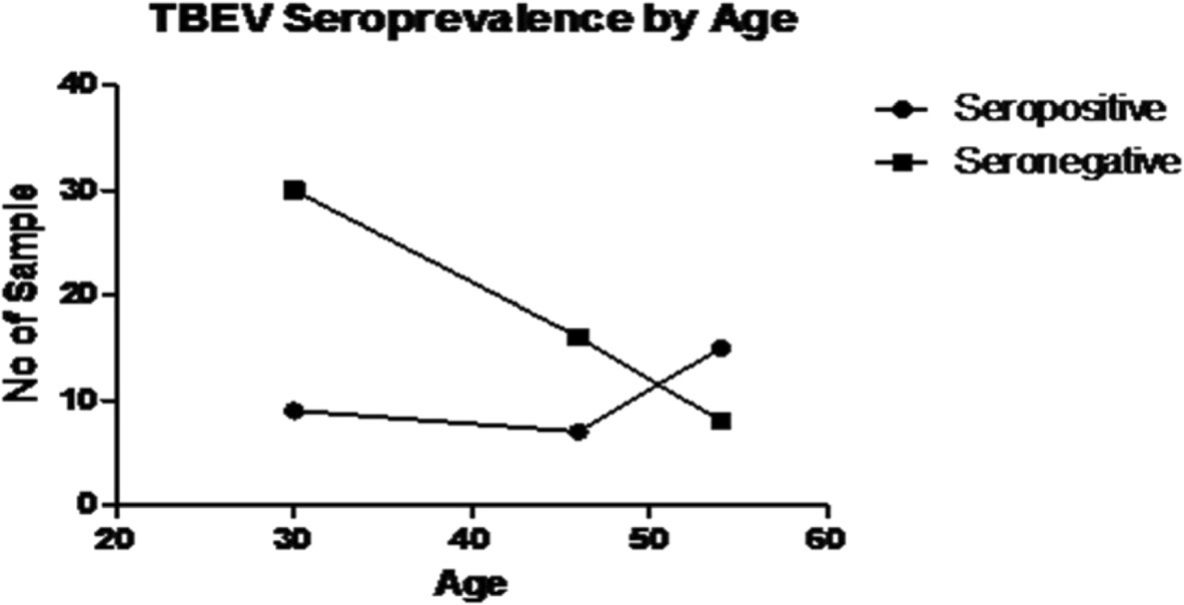
Correlation between seropositivity and age of studied participants. The figure showed the correlation between different age groups with seropositivity of tested samples

Seroprevalence data for TBEV and CCHFV are shown in Table 1. No positive result was obtained from any tested sample for anti-CCHFV IgG analysis. Nevertheless, serological evidence of TBEV IgG presence was identified among 31 (36.5 %) of 85 examined sera before excluding cross reactivity. We tested all 31 positive samples for anti-TBEV IgG for the presence of IgG against other three antigenically related flaviviruses including DENV, WNV and JEV to exclude possible false positive results due to cross reactivity. Our data showed that two TBEV IgG positive samples were positive for DENV IgG too. However, cross reactivity between TBEV IgG and WNV IgG was higher in that 17 positive samples for TBEV IgG were positive for WNV IgG too. We also found 11 sera with anti-JEV IgG antibodies (Table 2). Our overall data showed that there were two sera that reacted against all the flaviviruses. Seven of the 31 TBEV IgG positive samples were also positive for two flaviviruses. Only five (4.2 %) sera among the TBEV IgG positives were negative for all three flaviviruses. Twenty-two (70.97 %) of the 31 positive samples for anti-TBEV IgG were for subjects with tick-bite experience and none of them had a history of past vaccination for JEV. No statistically significant correlation was found between antibody prevalence and gender as presented in Fig. 2 (*P* = 1.0000 by Spearman correlation test). when seropositivity was analysed between different age groups, the outcome showed no significant difference (*P* = 0.5889). The highest reactivity towards anti-TBEV IgG ELISA was 68.4 % (13/19) in males aged 51–60 years and 50 % (2/4) in females in the same age group.

**Table 1:** Overall seropositive and seronegative results of TBEV and CCHFV

	General		Seropositive	Seronegative	Number of participants (*n* = 85)
	Gender	Male	27 (38.03 %)	44 (61.97 %)	71
		Female	4 (28.57 %)	10 (71.43 %)	14
TBEV		20–35	9 (23.08 %)	30 (76.92 %)	39
	Age (years)	36–50	7 (30.43 %)	16 (69.57 %)	23
		51–60	15 (65.22 %)	8 (34.78 %)	23
CCHFV			0 (0 %)	85 (100 %)	85

The table showed the number and percentage of seropositive and seronegative samples to the anti-TBEV (Tick-borne encephalitis virus) and anti-CCHFV (Crimean-Congo hemorrhagic fever virus) IgG ELISA, categorized by age and gender

**Table 2:** Cross reactivity of anti-TBEV IgG positive samples with DENV, WNV and JEV

Virus	Seropositive (#)	Seronegative (#)
DENV	2	29
WNV	17	14
JEV	11	20

The table showed the results for ELISA assays against DENV, WNV and JEV for all reactive samples against TBEV. The highest cross reaction can be seen between WNV and TBEV

## Discussion

We have shown the seropositivity for TBEV among a small group of farm workers in Peninsular Malaysia. Although our initial data showed that the prevalence of IgG against TBEV was 36.5 %, after testing the positive sera we found that only five (4.2 %) samples could be linked to the presence of TBEV. It was not surprising that there was cross reactivity between TBEV, WNV and DENV because they are all flaviviruses that share a complex antigenic relationship [17]. Our findings contradict previous studies by Thayan *et al*. [16] in which they stated that none of 600 tested samples of Malaysian patients with encephalitis carried TBEV antibodies. The difference is probably due to their selection of subjects who were not from a high-risk group and therefore did not meet the study requirements. To study a tick-borne disease, it is recommended to select subjects at high risk of tick bites, which is how most humans become infected [18]. Another reason for the difference between our findings and Thayan et al.’s could be the type of ELISA kit: the type of kits used in the two studies had different sensitivity and specificity. The TBEV IgG ELISA kit (Abnova® Corporation, Taiwan) that we used has a range of relative avidity index value of between 64 and 99 % in high avidity serum samples, and between 6 and 36 % in low avidity serum samples. Moreover, LGTV could be another reason for the detected seropositivity in our study [19]. LGTV is a Malaysian counterpart of TBEV with more than 80 % genome homology. Therefore, even using a virus neutralisation test, there could still be the chance of false positivity in the TBEV IgG ELISA results. Our findings show that 70.97 % of identified seropositive participants mentioned tick-bite experience and LGTV is also a tick virus, although without any clinical symptoms in humans. Our data showed that only five (4.2 %) sera were reactive against TBEV without cross reactivity with other tested flaviviruses, but there was uncertainty about the accuracy of the positivity of those samples because a virus neutralisation test was not performed. We were not able to do a virus neutralisation test using TBEV because this virus was not available in our laboratory and it is not an endemic virus in Malaysia. This was clearly one of the limitations of the study. We therefore strongly suggest considering a virus neutralisation test by collaborating with other certified laboratories when conducting future studies. CCHFV is claimed to be the most endemic tick-bornevirus with the widest geographical range compared with other tick-borne viruses [7]. Our data suggest that it is still not a threat to Malaysian farm workers. However, because there is evidence of sudden epidemics in nonendemic areas of different countries such as India, it is crucial to watch out for this infection. Although CCHFV is considered a potential occupational hazard among agricultural workers, a study by Sargianou *et al.* [20] showed a similar result of low seroprevalence (3.4 %) in Achaia, where citizens are almost entirely dependent on livestock farming. A serological survey conducted in Madagascar also showed very low occurrence of CCHFV infection in only 16 of 1995 tested workers [21]. However, higher prevalence data of 28.5 % have been reported in Iran due to CCHFV infection among livestock handlers and farmers [22]. A possible explanation for the low seropositive outcome for TBEV and the seronegative outcome of CCHFV between different studies and our study might relate to acaricides and rotational grazing systems. Seven of the eight farms in this study used acaricides and had a rotational grazing system. Acaricides are pesticides that kill ticks and mice. Rotational grazing combined with acaricide usage is a management practice to disrupt the parasitic life cycle and reduce the tick population on farms [23]. By keeping the pasture host-free to break down the natural life cycle of ticks, the system reduces the chances of tick survival by tick starvation [24]. According to Horst and Seifert, the larvae and nymph of ticks can be starved by keeping livestock away from a pasture for about 6 or 7 months. To eradicate adult ticks, the pasture must be host-free for about 14 or 15 months. The practice is most effective during hot and dry seasons [24]. However, in an area with strong winds, rotational grazing might be less efficient be- cause tick larvae may become wind-borne and carried away to neighbouring paddocks [24]. Tick management reduces the probability of infection among livestock and humans, leading to less positive results in seroprevalence studies. Methods of laboratory diagnosis are also closely related to outcomes obtained. The molecular method is useful for the early detection of a virus, while the serological method can only detect the presence of antibodies after the second week of infection. Detection of IgG only reveals previous virus infection and does not identify people who are currently infected [25]. There is a possibility that viruses are present in serum samples at an early phase of infection when antibodies are not detected by ELISA, and the analysis may therefore result in a false negative reading [26]. The outcome of this study suggested that seropositivity of TBEV did not increase with age and gender, as reported in another study in Lithuania [27]. Although the difference did not reach statistical significance, participants within the older age group (51–60 years) showed the highest seropositivity rate. This outcome was consistent with Mangarov et al.’s study in which they stated that the incidence and severity of TBEV were highest in 307 people over 50 years old [18]. This finding is most likely related to urbanisation, because it is common for younger citizens with a better educational background to migrate to urban areas [28]. Youngsters tend to move away from villages because they have a better opportunity to make a living in a working environment that is more convenient and less demanding than farming. This leads to the isolation of older people and youngsters with a lower educational background in rural areas, where farming is one of their main sources of income. The greater experience of older people means that they handle livestock more frequently than younger farmers. Since ticks feed from the blood of large mammals, frequent handling also increases the risk of a tick bite. Besides, antibodies accumulate and remain in the circulation for longer periods of time, which may lead to a higher titer in older individuals [29]. Certain groups of workers, such as farmers, veterinarians, shepherds and slaughterhouse staff, are considered at risk of zoonotic infections including TBEV and CCHFV because of the nature of their jobs [11]. Healthcare personnel are also at high risk because of exposure to infected patients’ blood and body fluids. This might be an alternative pathway for viral transmission, consistent with some investigations that have proven that infection by tick-borne viruses is more prevalent among high-risk than low-risk groups [22]. Nabeth *et al*. [30] suggested that direct contact with viraemic animals’ blood is the primary mode of animal-to-human transmission in high-risk groups. However, one study conducted in Iran suggested that CCHFV was not highly prevalent in high-risk professions [31]. As mentioned previously, there is no outbreak or reported cases of TBEV or CCHFV in Malaysia. This is in contrast to neighbouring countries, for example China, where the TBEV-Fe is endemic in certain parts of the country including Jilin Province, Inner Mongolia Province, Heilongjiang Province, Xinjiang Uygur Autonomous region and Yunan Province, and also Tibet [32]. In Japan, the first case of TBEV was reported in Hokkaido Prefecture. A phylogenetic study by Suzuki [5] showed that TBEV had been transmitted between Russia and Japan at least three times, most probably due to transmission of infected ticks by migratory birds travelling across the sea. Although there are no reported and confirmed cases of TBEV and CCHFV in Malaysia, there is no assurance that Malaysia will always stay a TBEV- and CCHFV- free country because many other factors may contribute to the viruses’ transmission, such as climate change and adverse environmental conditions including temperature and humidity [16].

## Conclusions

Our study showed a low seroprevalence for TBEV (4.2 %) among animal farm workers, after ruling out possible cross reactivities, and no correlation with age or gender. We also found no serological evidence of CCHFV transmission among Malaysian farm workers. Further studies involving a larger sample size, and also considering LGTV as a Malaysian counterpart of TBEV that might lead to false positivity, are highly recommended to provide sufficient evidence and reflect the true state of TBEV and CCHFV prevalence in Malaysia.

## Abbreviations

TBEV: Tick borne encephalitis virus
CCHFV: Crimean-Congo hemorrhagic fever virus
LGTV: Langat virus
IgG: Immunoglobulin G
IgM: Immunoglobulin M
ELISA: Enzyme-linked immunosorbent assay
DENV: Dengue virus
WNV: West Nile virus
TBEV-EU: Tick borne encephalitis virus European subtype
TBEV-Fe: Tick borne encephalitis virus Far-eastern subtype
TBEV-Sib: Tick borne encephalitis virus Siberian subtype
DVS: Department of veterinary services
